# La santé mentale des migrants entre textes juridiques internationaux et pratique clinique: quelle place pour la culture?

**DOI:** 10.11604/pamj.2023.44.98.37058

**Published:** 2023-02-20

**Authors:** Tareck Alsamara, Loubna Mouaatarif

**Affiliations:** 1 Prince Sultan University, Riyadh, Kingdom of Saudi Arabia,; 2 Institutions Médico-Sociales, Paris, France

**Keywords:** Santé mentale, migrants, droit international, culture, Mental health, migrants, international law, culture

## Abstract

Cette étude examine la santé mentale des migrants entre les textes juridiques internationaux et la pratique clinique. Elle met en lumière la réalité du droit à la santé mentale des migrants au niveau des textes juridiques internationaux. L'étude relie ensuite ce droit à la pratique nationale en France qui détermine la réalité de la pratique clinique de la santé mentale des immigrés. L'étude clinique vise à identifier l'adéquation des textes juridiques internationaux pour garantir ce droit en tant que partie intégrante des droits de l'homme. C'est l'individu dans sa singularité qui est au cœur de notre travail, toutefois notre regard multidisciplinaire abordera également les aspects socio-culturels, anthropologiques, environnementaux. En effet, imprégnés de réalités cliniques et sociales, nous nous demandons comment nier la dimension culturelle de toute interaction humaine donc à la base de la relation d'aide. Nous comprenons donc qu'il nous faut élargir notre cadre conceptuel et clinique/social à travers notre sensibilisation à l'anthropologie médicale clinique. La culture fabrique en partie l'individu, et ses comportements. Elle permet de mettre du sens sur les expériences qui surviennent dans la vie de chaque personne et de se préparer à ce qui pourrait arriver.

## Abstract (en)

This study examines the mental health of migrants under international legal texts and clinical practice. It highlights to what extent the right to mental health of migrants is guaranteed in international legal texts. It then relates this right to national practice in France. It determines practice guidelines addressing migrants' mental health. The purpose of this clinical study is to identify the adequacy of international legal texts to guarantee this right as an integral part of human rights. The individual in his or her singularity is at the heart of our work. However, a multidisciplinary approach will also address socio-cultural, anthropological and environmental factors. Indeed, steeped in clinical and social realities, we wonder how one can deny the cultural dimension of all human interactions and thus the basis of the helping relationship. We therefore understand that we need to broaden our conceptual and clinical/social framework through our awareness of clinical medical anthropology. Culture partly shapes the individual and his or her behaviour. It helps to make sense of the experiences that occur in each person's life and to prepare for what might happen.

## Commentaire

### Introduction

La migration a été définie par le secrétariat de l'Organisation mondiale de la Santé comme « Un processus de mouvement à travers une frontière internationale ou à l'intérieur d'un pays. C'est un mouvement de groupes de population qui comprend tout type de mouvement de personnes, quelle que soit sa durée, sa composition ou ses causes. Les migrants eux-mêmes se composent de groupes entremêlés de travailleurs migrants et de leurs familles, de personnes déplacées à long ou à court terme, et d'étudiants internationaux, de personnes déplacées à l'intérieur de leur pays, de demandeurs d'immigration et de réfugiés, de personnes retournant dans leur pays, de migrants en situation irrégulière et de victimes de la traite des personnes » [1].

Les raisons de la migration sont nombreuses et varient entre économiques [2] environnementales [3], climatiques [4] et parfois exil réfugiés politiques [5], qui dans tous les cas sont facteurs de fragilisation des individus vulnérables à la pression et à la maladie mentale [6]. Au contraire, la migration est un processus lourd de réinstallation et d'acculturation qui peut affecter négativement la santé mentale des migrants [7], et sur cette base, il faut veiller à la santé de ces personnes et à la nécessité de fournir et de garantir leur droit au traitement et aux services de santé [8]. De plus, les éléments identitaires et les dimensions culturelles de l'immigrant jouent un rôle fondamental dans sa santé psychologique et mentale [9]. L'intégration des immigrants contribue à améliorer leur santé psychologique et mentale [10].

A partir de notre réflexion juridico-clinique qui se base sur notre pratique d'accueil et d'accompagnement global d'un public vivant dans la précarité, nous comprenons que l'anthropologie médicale clinique est un outil de réflexion et de connaissance qui donne un aspect plus cohérent et plus ouvert sur l'interaction entre deux sujets soignants/soignés, aidant/aidés sans que l'appartenance à des codes de références culturelles différents voire totalement opposés ne viennent freiner la communication et la compréhension entre professionnels et migrants. Il s'agit d'éviter en effet deux écueils: celui du déni de la culture qui nie toute approche de la différence culturelle et ouvre la porte à tous les préjugés et dérapages ethnocentriques, comme celui de la tendance ethniciste qui explique tout par la culture, figeant l'autre dans sa culture dite d'origine. Dans ce travail, on propose d'abord d'introduire le cadre de l'anthropologie médicale, pour aborder ensuite quelques repères théoriques et méthodologiques de l'approche de la psychiatrie transculturelle qui en fait partie intégrante.

L'anthropologie médicale a incité les cliniciens à s'interroger sur les interactions réciproques entre (le dehors) la culture au sens anthropologique et (le dedans) le fonctionnement psychique de l'individu et droit d'asile. Elle s'intéresse aux relations entre soignés et soignants, aux représentations de la maladie du corps, à la formation des médecins. Les différentes formes d'expression et d'expériences de la souffrance ont été le sujet de nombreuses recherches en anthropologie médicale. La santé, la maladie, le malheur en tant qu'événement de la vie de chaque individu concerne toute l'humanité et prennent des formes d'expression et des réponses spécifiques et dépendantes de chaque société. Les réactions face à la souffrance peuvent être institutionnalisées et culturellement codifiées, témoignant ainsi de leur caractère de production humaine avec une raison d'être très précise [11]. La culture est une expérience intériorisée, au-delà de la dimension humaniste qui est à la base de notre réflexion juridico-psychosociale: l'idée est d'entrer le plus possible dans le monde de l'autre.

### L'absence d'un accord international spécialisé dans le droit à la santé des migrants

Le droit international se soucie de la santé et la considère parmi les droits de l'homme, [12,13] où il est stipulé dans l'article 25 de la Déclaration Universelle des Droits de l'Homme que le droit à la santé: « Toute personne a droit à un niveau de vie suffisant pour assurer la santé et le bien-être de lui-même et de sa famille, notamment en termes d'alimentation, d'habillement, de logement et de soins médicaux... »

Comme le stipule l'article 12 du Pacte international relatif aux droits sociaux et économiques: « Toute personne a le droit de jouir du meilleur état de santé physique et mentale ». Georges Devreux a été le premier à faire de la culture un levier culturel permettant l'élaboration psychique dans le travail thérapeutique. Cette idée inspirera de nombreuses structures d'accueil et de prise en charge des immigrés qui inclut pratique clinique et culture. Selon Georges Devereux l'ethnopsychiatrie se base sur deux éléments fondamentaux: la dimension universelle du psychisme humain et la manière dont il fonctionne ainsi chacun peut prétendre au même statut moral et scientifique si nous considérons leurs expressions culturelles comme des manières d'être et d'agir même si elles sont différentes. Si tout le monde tend vers l'universel c'est à travers les particularités de leur culture d'appartenance.

La culture est une expérience intériorisée qui fait partie intégrante de notre économie psychique et la manière structurée d'appréhender soi-même, et le monde à travers nos systèmes d'interprétations et de constructions de sens. La maladie n'échappe donc pas à ce codage culturel.

Au-delà de la dimension humaniste qui est à la base de toutes ces approches, l'idée est d'entrer le plus possible dans le monde de l'autre en acceptant d'apprendre du patient en conférant à son discours une légitimité, et non pas question de se spécialiser dans toutes les cultures. Il semble essentiel de percevoir les propres modèles explicatifs du “patient explanatory models of illness” (Kleinman) pour se donner les moyens d'honorer un contrat moral avec les patients en ayant pour objectif un échange humain et non machinique, et en le reconnaissant en tant que personne derrière ses symptômes. C'est ce que nous propose le modèle de l'anthropologie médicale clinique sur lequel notre travail s'appuiera.

### Le droit à la santé mentale humaine dans les documents internationaux spécialisés

L'article 5 de la Convention internationale sur l'élimination de toutes les formes de discrimination raciale reconnaît le droit à un niveau de santé adéquat quelle que soit l'origine ethnique; il stipule que «…Les États parties s'engagent à interdire et à éliminer la discrimination raciale sous toutes ses formes,...en particulier dans la jouissance des droits suivants: ...(e) ...(iv) le droit à la santé publique, aux soins médicaux et à la protection sociale sécurité et services sociaux;...».

L'article 7 du Pacte international relatif aux droits économiques, sociaux et culturels (1996) affirme que: « Les États parties au présent Pacte reconnaissent le droit de toute personne de jouir de conditions de travail justes et favorables, assurant notamment: ... b) des conditions de travail garantissant la sécurité et la santé...» comme le stipule expressément l'article 17 du Pacte: “les États parties au présent Pacte reconnaissent le droit de toute personne de jouir du meilleur état de santé physique et mentale susceptible d'être atteint.”

La Déclaration des droits des déficients mentaux réaffirme également la protection internationale du droit à un état de santé adéquat et à accorder une attention appropriée aux handicapés mentaux. Cette déclaration précise également que les personnes handicapées mentales doivent pouvoir bénéficier d'une assistance leur permettant d'atteindre le niveau le plus élevé possible en tant qu'individus.

Cette déclaration stipule que « Le déficient mental a le droit d'obtenir des soins et des traitements médicaux appropriés, ainsi qu'un degré d'éducation, de formation, de réadaptation et d'orientation qui lui permette de développer ses capacités et ses énergies dans toute la mesure du possible ».

Il est important de noter que les Principes pour la protection des personnes atteintes de maladie mentale et l'amélioration des soins de santé mentale (1991), doivent être fournis dans le cadre du système global de santé et de protection sociale; le principe 1 énonce: «Les libertés et les droits fondamentaux » à condition que: « Toute personne a droit aux meilleurs soins de santé mentale disponibles qui font partie du système de santé et de protection sociale ».

Au niveau régional, il convient de se référer au texte de l'article 16 de la Charte africaine des droits de l'homme et des peuples (1979) qui stipule expressément: « Toute personne a le droit de jouir du meilleur état de santé physique et mentale possible. Les États parties à la présente charte s'engagent à prendre les mesures nécessaires pour protéger la santé de leurs populations et assurer leur accès aux soins médicaux en cas de maladie ». Ces dispositions restent insuffisantes pour combler le vide juridique des textes internationaux. Parce que les textes internationaux sont importants, ils sont le premier moyen d'obliger les pays à promulguer une législation pertinente ou à adopter des politiques nationales qui garantissent ce droit.

### L'absence d'un accord international spécialisé dans la garantie de la santé mentale des migrants

Le droit des migrants à la santé mentale a une base juridique en vertu de l'article 28 de la Convention internationale sur la protection des droits de tous les travailleurs migrants et des membres de leur famille (1990) qui stipule expressément: « Les travailleurs migrants et les membres de leur famille ont le droit de recevoir tous les soins médicaux qui sont nécessaires d'urgence pour sauver leur vie ou pour éviter une atteinte irréparable à leur santé, sur la base de l'égalité de traitement avec les ressortissants de l'État concerné. Ils ne sont pas privés de ces soins médicaux d'urgence en raison d'une violation en matière de résidence ou d'emploi. Bien que la Convention ne parle pas de santé mentale, le concept de santé en général selon le texte de l'article inclut la santé mentale. L'article 70 du même document affirme que: « Les États parties prennent des mesures au moins égales à celles applicables à leurs ressortissants pour assurer que les conditions de travail et de vie des travailleurs migrants et des membres de leur famille en situation régulière sont conformes aux normes d'hygiène, de sécurité et de santé et aux principes de la dignité humaine ». Elle ne spécifie pas d'obligations strictes pour les États.

Par exemple les espaces solidarité insertion ont pour fonctions d'accueillir de manière anonyme, et gratuite un public de personnes démunies, de les informer et de les soutenir dans le cadre d'un accompagnement social et global notamment à travers des orientations vers les dispositifs de droits communs tant sur le plan social, médical, que juridique. L'équipe pluridisciplinaire est composée de deux éducateurs spécialisés, un moniteur éducateur, un animateur socio-culturel, un animateur et deux agents d'accueil et du chef de service. La prise en charge globale se déploie avec le travail en réseau avec des partenaires médicaux, sociaux et administratifs. Elle accueille ainsi de manière inconditionnelle, une population hétérogène d'adultes fragilisés. Ils sont souvent sans domicile fixe, migrants ou réfugiés, privés de travail ou bénéficiant des minimas sociaux.

Les histoires personnelles mettent en évidence l'existence d'une faille antérieure à la rupture sociale, d'une fragilité relationnelle ou affective réactivée par les difficultés économiques, professionnelles et pour la moitié d'entre elles dans un contexte migratoire. Des temps d'accueils (de 9h00 à 12h00) sont prévus dans le cadre d'une libre ouverture matinale autour de certaines prestations de base comme prétexte à la rencontre (petit déjeuner, espace hygiène, buanderie ...). Ce sont des moments propices à la création de liens. Les après-midi sont réservés aux accompagnements sociaux pour répondre aux demandes d'aide administratives (aide à la constitution de dossiers d'ouvertures de droits: couverture sociale: PASS, AME, CSS ...ou minimas sociaux: RSA, AAH ...), une aide à la recherche d'emploi (espace cyber) ou une aide à l'hébergement, ceci en intégrant dans la pratique quotidienne l'utilisation des dispositifs de droits communs déjà existant. Des permanences juridiques spécialisées au droit des étrangers pour répondre aux nombreuses personnes qui sollicitent conseil et espoir et le service accompagnement social pour constitution de dossiers d'ouvertures de droits. Ces structures proposent aussi en référence au cahier des charges des Espaces Solidarité Insertion (ESI) un travail de médiation et d'orientation thérapeutique quand cela est nécessaire et quand le moment est venu, faisant intervenir des équipes mobiles (psychiatrie, addictologie). La prise en charge globale se déploie ainsi avec le travail en réseau avec des partenaires pluridisciplinaires.

La population rencontrée dans ces accueils de jour connaît à notre sens un ensemble de mécanismes de rupture tant sur le plan symbolique que sur le plan des relations sociales, dans un contexte où s'intriquent des problèmes socio-économiques, psycho-affectifs, médicaux et au moins la moitié d'entre eux vient s'y greffer un déracinement culturel. En effet les flux migratoires liés à l'ouverture des frontières contribuent à modifier les origines géographiques et ethniques des populations à la rue. Primo arrivants, « sans papiers », réfugiés, statutaires, résidents, les situations sont toutes uniques et différentes, mais beaucoup connaissent des difficultés multiples ignorant leur(s) droit(s), ne connaissant pas les dispositifs d'aide, ils luttent au quotidien. La plupart investissent ces structures comme un lieu refuge.

### La nature du droit à la santé mentale des immigrants

Il est nécessaire de souligner que le droit à la santé mentale est différent des autres droits de l'homme. Il ne suffit pas de reconnaître légalement ce droit, car assurer ce droit nécessite des efforts supplémentaires de la part des États pour le garantir, car il ne n'exigent que la nécessité de le stipuler dans la législation nationale, mais plutôt la nécessité de garantir l'accès aux établissements de santé pour l'immigré [14], ainsi que la possibilité pour la société civile de contribuer à l'aide et à l'accompagnement des migrants, et l'implication des migrants dans le bénévolat contribue à leur intégration et à la préservation de leur santé mentale [15].

Deux facteurs s'associent dans la condition de l'immigré: l'écart culturel entre la société d'origine et la société d'accueil tant sur le plan de la confession, que des codes moraux, des traditions que de la mentalité et le bouleversement des liens affectifs. En effet, la migration possède en elle-même des potentialités traumatiques, du fait de la rupture du contenant culturel qu'elle implique. A l'origine de toute trajectoire migratoire il y a une situation de perte. Des deuils sont à élaborer: de personnes, de pays, de statut social. Migrer c'est bien sûr de laisser derrière soi: la famille, les amis, un métier, un statut social, la terre des ancêtres vivants et morts... Cela implique des renoncements, de la nostalgie, l'algie « du retour » donc quelque chose qui doit (ou devrait) être guéri par le retour. C'est un éloignement douloureux, une séparation mobilisatrice d'angoisse ou de mal être que l'individu est censé gérer en investissant beaucoup d'énergie psychique, psychosomatique, psychosociale. Les deuils sont parfois inacceptables. Les sentiments initiaux de douleurs intenses pour ce qui a été perdu, accompagné de désorganisation anxieuse dans un sentiment de détresse, solitude, abandon laissent progressivement la place à des affects dépressifs (et éventuellement à des défenses maniaques se traduisant par une minimisation ou une dénégation du changement survenu). Ce double mouvement de traumatisme et de perte vient redoubler celui qui est lié aux événements vécus.

S'échapper avec l'espoir difficile de revenir nécessite un travail plus difficile et plus complexe. La situation de la demande d'asile est encore plus compliquée, avec le risque de se voir refuser l'asile et d'être menacé d'expulsion. Il est facile d'imaginer comment cette menace résonne avec le sentiment d'anxiété intérieur. Les troubles observés sont souvent sensibles au codage culturel. Ce dernier se situe à un niveau ontologique (existence construite à partir de représentations culturelles) qui détermine l'étiologie du patient (le sens) et la logique thérapeutique. Une fois dans le pays d'accueil, nous constatons un décalage entre discours idéal sur l'asile et la réalité de l'opinion publique souvent critique des populations migratoires. La discrimination d'un public parmi les migrants, ceux réduits à des « sans-papiers » avec des lois qui handicapent encore plus ce qui sont en marge dans l'exclusion.

Le contexte socioculturel Le soignant l'aidant doit en effet entendre la souffrance psychique de l'autre et la souffrance liée à la migration: être à l'écoute de l'autre avec son parcours, sa souffrance, ses codes culturels, linguistiques, ses représentations religieuses, son statut social précaire ...etc. nous limitons nos propos à la population rencontrée dans le cadre de notre pratique dans ces accueils de jour. On retrouve à ce niveau tout ce qui touche à la scène sociale, à la réalité socioculturelle du patient/usager, tout ce qui légitimise son rôle social.

### La place des conditions de vie socio-économiques

Ils sont nombreux à être au chômage, sans aucune ressource, les uns résidents en France depuis plusieurs années: luttent pour faire renouveler leur titre de séjour, d'autres sont « sans papiers » provenant de pays défavorisés: ils vivent quotidiennement la menace de l'interpellation ou de l'expulsion. Les personnes en situation d'attente, pour lequel bien souvent le délai d'attente s'allonge. L'anxiété permanente engendre ou aggrave des pathologies physiques ou psychiques. La situation de logement est le plus souvent celle de l'hébergement. Beaucoup sont sans domicile fixe, ou peuvent même vivre dans des squats, et connaissent des passages à la rue. Dans le cadre de notre travail d'accès aux droits, une permanence juridique hebdomadaire ouverte à tous est assurée par une équipe de conseillers juridiques et avocats spécialistes du droit des étrangers est mise en place dans nos locaux afin d'orienter les usagers ou de les aider dans le cadre de leur démarche de régularisation de séjour constatant les demandes récurrentes. En effet, beaucoup ignorent leurs droits tant en matière juridique qu'en matière de soins. Les restrictions, légales et administratives aux principes du droit à la santé pour les étrangers sont autant de contraintes s'opposent à une prise en charge thérapeutique efficace. Les facteurs psychosociaux associés aux problèmes de santé mentale des migrants doivent être considérés en tenant compte de trois dimensions: le contexte, la culture et la communauté. L'intégration de ces trois dimensions permet de saisir les configurations de facteurs de vulnérabilité ou de protections qui vont interagir de façon dynamique. Les migrants sont porteurs de plusieurs cultures qui envahissent et structurent tous les aspects de la vie sociale. L'équilibre entre ses cultures, celle de leur pays d'origine, celle associée à une identité de migrant et celle du pays hôte doit s'établir de façon différente dans divers champs sociaux. On peut par exemple adopter les comportements et les valeurs du pays hôte dans le domaine du travail et conserver les valeurs et façon de faire de son pays d'origine en ce qui concerne les relations familiales. Ses cultures s'enracinent dans un contexte spécifique qui est changeant. A ce niveau, le contexte socio-économique joue un rôle de premier plan. En effet l'approche interculturelle ne doit pas être un alibi des difficultés et des problèmes qui relèvent davantage de la précarité sociale et économique que des différences culturelles. Le médiateur culturel est celui qui révèle et explique de manière bilatérale les représentations culturelles, les valeurs et les normes qui y sont rattachées.

### Les décodages linguistiques

Bon nombre de migrants ou réfugiés pour des raisons variées dues au contexte migratoire ou d'exil, ne maîtrisent pas suffisamment la langue française pour permettre une communication efficace dans la relation soignant/soigné, aidant/aidé.

La barrière de la langue est l'obstacle manifeste dans la communication avec certaines personnes migrantes ou d'origine étrangère que nous rencontrons. La fonction du médiateur se fait au niveau linguistique et permet de faire le pont en reliant deux mondes culturels. À partir de la référence de principes et d'expérience à la psychanalyse qui fait porter l'attention aux modalités du discours, à ses significations latentes, où des forces psychiques, fantasmes. Il parait clair que les simples traductions négligent ou suppriment un ensemble de signes, d'indices, de troubles de l'expression ou d'autres symptômes qui sont des éléments révélateurs du fonctionnement psychique et de ses avatars. En effet, l'usage de la langue maternelle par l'introduction d'un médiateur-interprète, en même temps qu'il facilite l'expression des affects permet aussi une sorte d'aller et retour entre les dimensions psychologiques et culturelles. Comme l'explique Rechtman dans « L'intraduisible culturel » il faut souligner la qualité de l'interprète: on récusera les membres de la famille ou proche, lors de la conduite de l'entretien l'interprète ne doit pas être envahi par les représentations du patient, susceptible de faire écho à son histoire personnelle: emploi du pronom “je” à la première personne pour permettre aussi le travail de traduction en accélérant l'échange nécessitant un travail de distance et d'analyse des mouvements internes vécus par l'interprète culturel, et l'évaluation de la relation interprète/patient.

### Les changements culturels qui accompagnent l'acculturation

Nous ne pouvons demeurer indifférents devant les phénomènes d'acculturation qui renvoient à un contexte politique. Divers modèles existent, par exemple, le contexte français d'idéologie républicaine marqué par la citoyenneté universelle, le lien historique particulier avec la majorité de la population immigrée, etc. Contrairement à la situation au Canada, où le multiculturalisme prône et une immigration essentiellement sédentaire [16]. Même si les processus d'acculturation sont comparables, les pratiques françaises et canadiennes sont différentes. L'institution en mettant en place des moyens, des outils, des programmes permettant d'améliorer la relation interculturelle, souhaite accompagner l'autre dans les processus d'acculturation. Ce contexte socio culturel (non exhaustif, il ne s'agit là que de poser quelques repères pour penser notre travail) traits socioculturels, familiaux, professionnels, personnels, évoluent, s'accentuent, s'amoindrissent selon les contextes au fil des histoires personnelles, des rencontres réalisées, des choix posés, toujours avec l'histoire des relations entre peuples en toile de fond. C'est à partir de là que peuvent intervenir les aidants/soignants. Le choc culturel contrairement aux idées reçues peut être porteur d'apprentissage et source d'entente à condition qu'il soit bien analysé ...

### Le processus d'acculturation

Dans la tradition des études sur l'immigration, une hypothèse fondamentale est que les immigrants ont des problèmes d'adaptation (psychologiques et sociaux). Dans une collection d'œuvres de Berry John, la relation entre l'acculturation et l'adaptation psychologique est discutée. Redfield, Linton et Herskovits définissent l'acculturation comme une série de changements culturels résultant d'un contact continu et direct entre deux groupes culturels distincts: le groupe dominant et le groupe non dominant, le groupe d'acculturation [17,18]. Plusieurs changements psychologiques peuvent être observés pendant et après l'acculturation: au niveau de l'identité personnelle et ethnique, des attitudes, des compétences, de la motivation, certains de ces changements ont des effets positifs, comme l'amélioration des conditions économiques, l'éducation médicale, d'autres se manifestent dans la forme de problèmes psychologiques comme le stress d'acculturation. Deux questions se posent: est-il important de conserver son identité culturelle? Est-il important de rechercher des relations (sociales, économiques, etc.) avec d'autres groupes de la société (coutumes, langue, religion)? Ces questions peuvent être répondues par oui ou par non, conduisant à quatre modes d'acculturation: assimilation, il existe deux types d'assimilation: l'une se fait par assimilation du groupe non dominant au groupe dominant et l'autre se fait par assimilation. Par l'intégration de plusieurs groupes dans une nouvelle société homogène - une intégration dans laquelle les individus sont plus ou moins clairement impliqués. Dans ce cas, la personne conserve son identité et d'autres spécificités culturelles (langue, habitudes alimentaires, etc.) tout en participant à la nouvelle société. Séparation lorsque l'individu ne recherche pas une relation avec la communauté dominante qui marquera son identité culturelle. - isolement: lorsqu'un groupe dominant oblige un groupe non dominant à maintenir son identité culturelle. Berry parle de quatre groupes: les immigrés: ils ont majoritairement une attitude positive vis-à-vis de l'immigration car c'est leur choix. Les réfugiés ont moins de liberté de choix car ils doivent quitter leur pays pour survivre (guerre). Les sédentaires, les autochtones vivant dans les territoires autochtones sont dominés par des groupes d'immigrants ethniques plus forts issus d'anciens immigrants, avec plus ou moins leur participation volontaire à la vie de la société d'accueil. Enfin, le stress est un concept utilisé pour identifier l'état physiologique dans lequel un organisme réagit aux conditions environnementales: *(Stressor)* passe par un processus d'ajustement *(coping)* afin de s'adapter de manière satisfaisante à la situation proposée. Le stress d'acculturation provenant du processus d'acculturation lui-même se manifeste par des problèmes de santé mentale: confusion, dépression, anxiété, marginalisation, problèmes d'identité, qui sont plus fréquents chez les populations immigrées: réfugiés et autochtones.

### Conclusion

Notre étude souligne l'idée majeure que le droit international ne comprend pas d'accords internationaux solides pour protéger la santé mentale des migrants. Nous soutenons également la nécessité d'adopter une approche culturelle dans la prise en charge des migrants, afin de maintenir leur santé mentale comme partie intégrante de la santé en général. En effet, les personnes migrantes, réfugiées, exilées demeurent les plus vulnérables de la société puisqu'elles portent en elles des blessures invisibles relevant de la santé mentale plus que de la santé physique. Elles ont en effet été confrontées à la guerre, ont subi des catastrophes, ont vécu des événements traumatisants. Elles sont donc plus exposées à l'anxiété, la dépression, au trouble du stress post-traumatique trop souvent détecté tardivement ou parfois non détecté. Les difficiles parcours migratoires de ces familles ainsi que leur intégration dans le pays d'accueil les confrontent trop souvent à des conditions précaires voire extrêmes qui mettent à mal leur santé mentale. Nous déplorons alors les difficultés rencontrées par les établissements de santé dans la prise en charge des migrants, compte tenu des aspects culturels manquants qui favorisent pourtant l'intégration et l'acculturation. Il est donc important que toute personne, y compris les migrants, les réfugiés, jouissent du droit à la santé (mentale et physique) en développant des centres de santé solides et complets comportant la prise en compte des différences culturelles avec le recours à un interprète pour leur permettre l'expression de leur histoire et de leurs souffrances. Pour finir, nous souhaitons rappeler que les migrants peuvent contribuer positivement au développement de la société. Pour cela, il faut les aider à maintenir ou à retrouver leur plein potentiel en garantissant leur santé physique et mentale.

## Remerciements

Tous les auteurs de cet article tiennent à remercier le laboratoire de recherche sur la gouvernance et la conception des politiques (GPDRL) de l'Université Prince Sultan (PSU) pour son soutien financier et académique pour mener cette recherche et la publier dans le Journal respecté.
